# Weight-Bearing Status After Peri-Prosthetic Proximal Femur Fracture Open Reduction and Internal Fixation (ORIF) or Revision Arthroplasty: A Clinical Audit

**DOI:** 10.7759/cureus.90805

**Published:** 2025-08-23

**Authors:** Owen Mitchell, Peter Ward, Kaloian Petrov

**Affiliations:** 1 Trauma and Orthopaedics, Dorset County Hospital, Dorchester, GBR; 2 Orthopaedics, Shepton Mallet Hospital, Shepton Mallet, GBR

**Keywords:** open reduction and internal fixation, peri-prosthetic proximal femur fracture, revision arthroplasty, vancouver classification system, weight-bearing

## Abstract

Background

Peri-prosthetic proximal femur fractures (PPFFs) are becoming increasingly common due to the rising number of total hip arthroplasties (THAs) in ageing populations. These complex fractures often require either open reduction and internal fixation (ORIF) or revision arthroplasty. Postoperative weight-bearing (WB) status plays a critical role in recovery, yet there is considerable variability in clinical practice.

Objectives

This audit aimed to evaluate the documentation and implementation of postoperative WB protocols following ORIF or revision arthroplasty for PPFFs. It assessed adherence to British Hip Society best practice guidelines, reasons for deviations, and rates of fixation failure.

Methods

A retrospective single-cycle audit was conducted at a UK District General Hospital between January 2023 and June 2024. Data was collected from the Hip Fracture Registry. Inclusion criteria were patients with closed PPFFs around THA or hemiarthroplasty; exclusions included fractures involving intra-medullary devices or knee prostheses. Patients were classified using the Vancouver classification (types A-C). Data points included fracture type, treatment modality, postoperative WB status, rationale for WB decision, and fixation outcomes.

Results

Thirty-eight patients met the inclusion criteria: four (10.5%) had type A fractures, 28 (73.7%) type B, and six (15.8%) type C. Twenty-eight (73.7%) patients underwent surgical intervention (19 ORIF; nine revision arthroplasty), while 10 (26.3%) were managed conservatively. Of the surgical group, 22 (78.6%) were mobilised as full weight-bearing (FWB), four (14.3%) were limited weight-bearing (LWB), and two (7.2%) were non-weight-bearing (NWB). LWB was most often attributed to the intraoperative identification of poor bone stock; NWB was associated with medial calcar instability or metastatic involvement. Only one (3.5%) patient experienced fixation failure, with conservative management adopted due to comorbidities. A notable finding was the lack of detailed documentation for WB restrictions in LWB and NWB groups, which posed challenges for postoperative rehabilitation.

Conclusions

This audit demonstrates that while the majority of PPFF patients were managed in accordance with national guidance promoting early FWB, a significant proportion received restricted WB without adequate justification. The low rate of fixation failure supports the feasibility of immediate FWB in appropriately selected patients. However, the absence of clear functional instructions in many cases highlights a need for improved communication and standardised documentation. Further multi-centre studies are required to establish robust, evidence-based postoperative protocols for this growing patient population.

## Introduction

Peri-prosthetic proximal femur fractures (PPFFs) are increasingly encountered in orthopaedic practice, largely due to the growing number of total hip arthroplasties (THAs) being performed in ageing populations [1]. In the United Kingdom, there is currently a 3.5% risk of PPFF after a THA, which is projected to rise to 4.6% in the next 30 years [2]. These fractures, occurring around existing prosthetic implants, present unique surgical challenges due to the complexity of bone-implant interactions, reduced bone stock, and the necessity of individualised treatment strategies [3]. The management of PPFFs often involves either open reduction and internal fixation (ORIF) or revision arthroplasty, depending on fracture classification, prosthesis stability, and patient factors [4].

Postoperative weight-bearing (WB) status plays a pivotal role in recovery following both ORIF and revision procedures for PPFFs. While early mobilisation has been associated with improved functional outcomes and reduced medical complications, WB restrictions are frequently imposed due to concerns over fixation stability and risk of implant failure [5]. Guidelines published by the British Hip Society advise surgeons to aim to allow full weight-bearing (FWB) in the immediate postoperative period [6]. However, there is considerable variability in clinical practice, with decisions often based on surgeon experience or institutional norms rather than standardised, evidence-based guidelines [7]. This study utilised the Vancouver classification of PPFFs in order to help categorise the patients. This classification helps guide surgeons with the decision-making process in the management of each specific patient, between conservative management, ORIF, and revision arthroplasty [8]. The system categorises the fracture based on its location relative to the femoral stem, the stability of the implant, and the quality of the surrounding bone stock [8]. Type A fractures are located around the peri-trochanteric regions, type B is for fractures around or just below the stem, with a further subdivision based on the stability of the implant and surrounding bone stock, and type C fractures are well below the stem [8].

This clinical audit aims to evaluate the documentation and implementation of postoperative WB protocols in patients undergoing ORIF or revision arthroplasty for PPFFs at a District General Hospital in the United Kingdom. This audit will assess adherence to current best practices, explore the barriers to implementing FWB in the immediate postoperative period, and assess the fixation failure rate after PPFF ORIF or revision arthroplasty.

## Materials and methods

This study was conducted at the Department of Trauma and Orthopaedics of Dorset County Hospital, located in Dorchester, United Kingdom. The approval for the audit was granted by the Clinical Audit Department of Dorset County Hospital in 2024 (audit number: 6102). Data was collected retrospectively from the department's Hip Fracture Registry for the period of January 2023 to June 2024. The inclusion criteria were patients who presented with closed PPFFs with a pre-existing THA or hemiarthroplasty. The exclusion criteria omitted any patients presenting with fractures around a previous femoral intra-medullary device, dynamic hip screw, total knee arthroplasty, or unicondylar arthroplasty.

The patients were then classified using the Vancouver classification system and were placed into fracture group A, B, or C [8]. The patients' notes were then studied, with relevant data collection points being the classification of the fracture, the treatment option chosen, why this treatment option was decided, immediate postoperative WB status, why this WB status was decided, and any further failure of fixation.

The data was then gathered, analysed, and presented at a local orthopaedic educational meeting to key stakeholders (orthopaedic surgeons and resident doctors) about the importance of aiming for FWB in the immediate postoperative period after these interventions.

## Results

Following the application of the selection criteria, this audit identified 38 patients who were admitted to the orthopaedic department. Of these patients, there were four (10.5%) with Vancouver A fractures, 28 (73.7%) with Vancouver B fractures, and six (15.8%) with Vancouver C fractures.

Twenty-eight (73.7%) patients underwent surgical intervention, 19 (50%) had ORIFs, and nine (23.7%) had revision arthroplasty. The rest of the patients were treated conservatively due to the undisplaced nature of the fractures, the patients being unfit for surgery, or the patients passing away before surgery could be undertaken.

Of the patients who underwent surgical intervention (ORIF or revision arthroplasty), operative documentation indicated that 22 (78.6%) were allowed to be FWB immediately after the operation. Four (14.3%) patients were given limited weight-bearing (LWB) status immediately postoperatively, and two (7.2%) were non-weight-bearing (NWB).

For the patients who were not advised to be FWB in the immediate postoperative period, the patients' operation note was reviewed for justification. The most common reason for LWB was due to poor bone quality found intraoperatively. For the NWB patients, the main reasons were due to the instability of the medial calcar and the presence of significant metastatic deposition in the femoral shaft. 

From the data collected in this audit, there was one failed fixation, who was FWB after their operation and subsequently failed within six weeks. They were subsequently treated conservatively due to the patient being unfit for further intervention. See Table 1 for full results.

**Table 1:** Retrospective data gathered for peri-prosthetic proximal femur fractures arranged by their Vancouver classification, treatment, and weight-bearing status after surgical intervention ORIF: open reduction and internal fixation

Vancouver classification	Number of fractures	Number of fractures managed conservatively	Number of fractures managed with ORIF/revision arthroplasty	Number of full weight-bearing after surgery	Number of limited weight-bearing after surgery	Number of non-weight-bearing after surgery	Number of fixation failures after surgery
A	4 (10.5%)	4 (100%)	0 (0%)	0 (0%)	0 (0%)	0 (0%)	0 (0%)
B	28 (73.7%)	4 (14.2%)	24 (85.7%)	20 (83.3%)	2 (8.3%)	2 (8.3%)	1 (3.5%)
C	6 (15.8%)	2 (33.3%)	4 (66.6%)	2 (50%)	2 (50%)	0 (0%)	0 (0%)
Total	38 (100%)	10 (26.3%)	28 (73.7%)	22 (78.6%)	4 (14.3%)	2 (7.2%)	1 (3.5%)

## Discussion

This audit has found that 22 (78.6%) patients were FWB after their operation, which is the current recommendation from the British Hip Society guidance [6]. Immediate postoperative FWB has been shown to reduce the risks of complications associated with prolonged immobility, such as deep vein thrombosis, pulmonary embolism, and muscle atrophy [9]. In addition to this, the benefits of allowing FWB include improved functional recovery, mobility, and return to independence at home [10]. This not only benefits the patients' quality of life but also helps to reduce the length of stay in hospital and the healthcare costs involved with managing these complex injuries and frail, comorbid patients. Furthermore, allowing FWB postoperatively will increase the physiological stress on the fracture site and thereby stimulate healing and remodelling of the bone for patients who were managed with ORIF [11].

There is a lack of evidence for the benefits of FWB in our patient cohort. However, a study by Smith et al. demonstrated that immediate FWB post-locked plate fixation for peri-prosthetic distal femur fractures achieved a 93% union rate and a 73% return to pre-injury ambulation by one year, suggesting this strategy is both feasible and effective [12]. 

Despite the advantages, immediate FWB is not without risks. Mechanical failure of the fixation construct, especially in the presence of medial comminution, poor bone stock, or suboptimal implant positioning, remains a concern. Studies have identified medial support loss as an independent predictor of failure in locked plate constructs [13]. In revision arthroplasty, failure to achieve sufficient primary stability, particularly in cases with extensive bone loss, can compromise the integrity of the construct under full loading conditions [14]. This study found a very low rate of fixation failure within one year, just one (3.6%) of the studied cohort. This figure suggests that patients are not being instructed to FWB if it is not appropriate.

The data found in this audit showed that four (14%) patients were advised to have LWB on the operative leg; this was primarily due to poor bone stock found intraoperatively. However, one issue that was highlighted was the lack of description for specific functional or WB constraints as part of the LWB advice. This made it very difficult for the patient and physiotherapists to know what specific movements were safe to carry out. This could prolong hospital stays and possibly lead to patients WB too much through the leg and jeopardise the structural integrity of the fixation [9].

In order to improve this, the British Orthopaedic Association has advised for thorough patient-specific documentation for the clinical rationale behind LWB, to quantity the functional or distance restrictions placed on the patient (it should not be documented by weight or percentage of bodyweight), and to specify the length of time that the patient should have the WB restrictions in place [15].

Immediate FWB following ORIF or revision arthroplasty for proximal PPFFs presents both clear advantages and inherent risks. It can enhance recovery and reduce immobility-related complications in appropriately selected patients. However, careful assessment of fixation stability, fracture morphology, and patient factors remains critical to avoid implant failure. There is a growing need for prospective, multi-centre trials to establish standardised protocols and clarify the indications for immediate FWB in this patient population.

Limitations

There are a few limitations identified in this study. Firstly, it is a single-centre study from a District General Hospital with an 18-month data collection period, with a relatively small number of patients included. This could mean that the reliability of the data collected may not be applicable or representative at a regional or national level. Furthermore, this study identified a lack of specific evidence in the literature for the risks and benefits of FWB immediately postoperatively, indicating the need for a larger study or trial to help standardise protocols. This audit also did not compare the rates of FWB after revision arthroplasty with ORIF to see if there was any discrepancy between these two surgical options.

## Conclusions

This clinical audit highlights the current variability in postoperative WB protocols following ORIF or revision arthroplasty for PPFFs. While the majority of patients (78.5%) were appropriately permitted FWB in line with British Hip Society guidance, a substantial proportion (22.5%) were managed with LWB or NWB status, often without detailed documentation or clear functional parameters. This inconsistency poses challenges for rehabilitation teams and may hinder optimal recovery. The audit supports growing evidence that immediate FWB, when fixation is stable and patient-specific factors are considered, is both safe and beneficial in enhancing functional recovery and reducing complications associated with immobility. Importantly, the low fixation-failure rate observed (3.5%) reinforces the feasibility of early mobilisation in appropriately selected cases. However, the lack of standardised, evidence-based guidelines and insufficient intraoperative documentation of WB decisions remain barriers to consistent best practice. To improve patient outcomes and clinical efficiency, it is essential to implement more rigorous documentation of WB instructions, including clearly defined functional limitations and timelines. Moreover, multi-centre studies with larger cohorts are required to validate these findings and develop robust protocols that can guide postoperative management across institutions.
